# Urinary and Renal Derangements

**Published:** 1885-10

**Authors:** 


Urinary and Renal Derangements and Calculous Disorders—Hints on Diagnosis and Treatment. *By* Lionel S. Beale, m. d. *Pp. 356. Philadelphia: P. Blakiston, Son & Co. Chicago: W. T. Keener.*

This work comprises much that the author has already written upon this subject, together with the more recently developed facts.

The first 68 pages are devoted to the consideration of the physiological constituents of the urine and their proportions in normal and pathological states. The clinical features accompanying the variations from the normal standard are briefly set forth, and the indications for treatment; a short statement of the action and special application of such remedies as the author has found most efficient is given.

The second part, pp. 68-209, treats of the urinary deposits, their pathological significance, and the appropriate treatment of the conditions upon which they depend.

The third part treats of substances in solution, not found in healthy urine ; albumen, its significance in the various forms of kidney disease, and the nature of the various structural tissues giving rise to its presence, together with the symptoms they produce, and the points of differential diagnosis ; also hints as to the measures to be taken in combating these pathological processes. Sugar, bile and alkapton are similarily considered under the same heading.

The remainder of the book, pp. 309–356, is given to the consideration of urinary calculi and calculous disorders—the conditions under which calculi are formed, the modes of their formation, and the treatment, preventive, pallative and curative.

The book contains so much that is useful that it is to be regretted the author has not arranged an alphabetical index. The table of contents only partially supplies the deficiency.

